# Vortioxetine for Cognitive Enhancement in Major Depression: From Animal Models to Clinical Research

**DOI:** 10.3389/fpsyt.2019.00771

**Published:** 2019-11-06

**Authors:** Djamila Bennabi, Emmanuel Haffen, Vincent Van Waes

**Affiliations:** ^1^Department of Clinical Psychiatry, INSERM, CHU de Besançon, Neurosciences, University Bourgogne Franche-Comté, FondaMental Foundation, Creteil, France; ^2^Laboratory of Integrative and Clinical Neuroscience, University of Bourgogne Franche-Comté, Besançon, France

**Keywords:** major depressive disorder, vortioxetine, cognition, humans, animals

## Abstract

**Objectives:** Vortioxetine has already shown its efficacy in the acute and long-term treatment of major depressive disorder (MDD) and its potential interest in the prevention of relapse. The aim of this study was to review the current status of knowledge regarding its cognitive effects.

**Methods:** We conducted a review of key data obtained from preclinical behavioral models and clinical trials in MDD focusing on vortioxetine-induced cognitive changes.

**Results:** In animals, acute and chronic administration of vortioxetine improves performance on objective measures that cover a broad range of cognitive domains. In human, vortioxetine appears to be a useful treatment option in MDD patients with cognitive dysfunction.

**Conclusion:** Vortioxetine constitutes a promising treatment for treatment of cognitive impairment in MDD, but its place in the therapeutic armamentarium still needs to be determined.

## Introduction

Major depressive disorder (MDD) is a widespread psychiatric illness, associated with reduced productivity, deterioration in quality of life, and important morbidity and mortality (1, 2). MDD is one of the main causes of global burden by disability-adjusted life-years and may become the leading cause of disease burden by 2030, according to WHO (3). Cognitive deficits are often present in the clinical presentation of MDD and constitute a substantial component of the personal, familial, and social burden of the disease (4, 5). These deficits cover fundamental cognitive domains, such as psychomotor speed, attention, learning and memory, executive functioning, and processing of emotionally valenced stimuli (6).

In the acute phase, cognitive problems represent a key mediator of health outcomes in subsets of patients. They are frequent residual manifestations, following remission of the current episode, and constitute good predictors of functional and social outcomes for depressed patients (7). This observation is of particular relevance if taken into consideration how psychosocial functioning impairments, notably decrements in work productivity, account disproportionately for the overall costs attributable to MDD (8, 9). Considering that one of the main objectives of treatment is achieving functional recovery, there is growing interest in therapeutic strategies targeting cognitive function.

Vortioxetine is an antidepressant with a multitarget profile that embraces several pharmacological actions: inhibition of the serotonin (5-HT) transporter (SERT) and direct modulation of receptor activity (i.e., partial agonism at 5-HT1B receptors, agonism at 5-HT1A, and antagonist properties at 5-HT1D, 5-HT3, and 5-HT7 receptors) (10). There is accreting mechanistic evidence that vortioxetine can also indirectly modulate dopaminergic, noradrenergic, histaminergic, cholinergic, GABAergic, and glutamatergic systems. Its efficacy and safety in patients suffering from moderate to severe MDD are supported by positive short-term (6- to 12-week) randomized, placebo-controlled trials and one positive randomized, double-blind, 52-week relapse prevention trial (11). In pooled analyses of randomized trial data, higher rates of positive response and successful remission following treatment with vortioxetine were observed when compared with placebo, which supported its approval by the US Food and Drug Administration and the European Medicines Agency (12). Moreover, vortioxetine is apparently well tolerated. Indeed, incidence of secondary effects on body weight, sleep, or sexual functioning seems to be low (10). Besides its efficacy in the acute and long-term treatment of MDD and its interest in the prevention of relapse, vortioxetine’s unique pharmacological profile makes it particularly interesting regarding therapeutic effects on cognitive impairment. The efficacy of vortioxetine in the management of MDD has been mainly linked to its action on synaptic serotonin levels *via* inhibition of SERT, but its cognition-enhancing properties are hypothesized to be also mediated by other mechanisms. The goal of this review is, therefore, to bring together and to summarize key data obtained from preclinical behavioral models and clinical trials in MDD focusing on vortioxetine-induced cognitive changes. Limitations and perspectives are then discussed.

## Methods

Screening for studies on the cognitive effects of vortioxetine in preclinical and clinical settings was conducted on PubMed, PsycINFO, and Google Scholar databases. No limits were applied to publication year. Search for articles was based on the following keywords using Boolean operators: (1) for preclinical studies: “vortioxetine”, “rats,” “mice,” “depression,” “cognition”; (2) for clinical studies: “depression,” “cognition,” “vortioxetine.” Inclusion criteria for clinical studies were as follows: (a) publication in English, (b) inclusion of unipolar depressed patients, (c) controlled clinical trial or meta-analysis, and (d) measures of cognition as primary or secondary outcome. Our search criteria yielded 28 references. The reviewed studies are listed in **Tables 1**–**3**.

### Animal Studies

#### Vortioxetine Decreases Depression-Related Behaviors in Rodents: an Effect Possibly Mediated by Direct Activity At 5-HT Receptors

In the rat, vortioxetine has a dose-dependent antidepressant-like effect in the forced swim test (FST) in an animal model of depression (Flinders Sensitive Line rats, minimal effective doses: 7.8 mg/kg subcutaneously [s.c.], administered 24, 6, and 1 h before the FST procedure) and anxiolytic-like effects in social interaction and conditioned fear tests (Sprague–Dawley rats, minimal effective doses: 2 [orally {p.o.}] and 3.9 [s.c.] mg/kg administered acutely, respectively) (40). Further microdialysis experiments carried out in the same study revealed that vortioxetine (2.5–10 mg/kg s.c.) increases extracellular 5-HT, dopamine, and noradrenaline in the medial prefrontal cortex as well as in the ventral hippocampus, especially for the highest dose tested. Subchronic administration of vortioxetine (5 mg/kg per day for 3 days; minipump implanted s.c.) also significantly increased extracellular 5-HT in the ventral hippocampus (40). Another study (chronic administration) in healthy middle-aged female mice indicated that both 1- and 3-month treatment with therapeutic doses of vortioxetine decreases the percentage of immobility in the FST (22). This is in accordance with data showing in healthy mice that acute and repeated dosing of vortioxetine produces more pronounced anxiolytic- and antidepressant-like activities than fluoxetine (41). This effect is associated with increased neurogenesis levels in the hippocampus (41), which are hypothesized to be one of the neurobiological mechanisms of action of antidepressant drugs (42).

Numerous studies have been conducted in various animal models of depression. For instance, it has been shown that chronic vortioxetine treatment reverses the increased time spent immobile in the FST observed in tryptophan depletion (43) or progesterone withdrawal (44) models. In line with these results, vortioxetine, fluoxetine, and S-ketamine induce in Flinders Sensitive Line rats an acute antidepressant-like activity (FST), but only S-ketamine has sustained effects (19). Unlike vortioxetine, the antidepressant-like responses of fluoxetine and S-ketamine are abolished by 5-HT depletion (44). Interestingly, using the SERT Met172 mouse model, which disrupts the high-affinity interactions of many antidepressants with SERT, Nackenoff and collaborators (45) showed that the antidepressant actions of vortioxetine may be SERT-independent. Indeed, vortioxetine was still able to enhance mobility in the tail suspension test and in the FST, to reduce consumption latency in the novelty-induced hypophagia test, and to promote proliferation and survival of subgranular-zone hippocampal stem cells, despite less interaction with SERT in these mice (45). Exposure to early stressful life events (in utero) is also an animal model of depression (46). It has been reported that single, chronic, and concomitant use of vortioxetine, dapoxetine, and fluoxetine has antidepressant and anxiolytic effects in prenatally stressed rats (47). 

It is worth noting the critical role that affective biases apparently play in the onset and development of depression. Refsgaard and collaborators (48) used a recently developed affective bias test for rats to evaluate the modulation of affective biases by vortioxetine, sertraline, duloxetine, and the α2 adrenoceptor antagonist idazoxan. Vortioxetine and idazoxan induced the largest effects, and at all tested doses, there were significant positive biases as outcome (48).

Altogether, these observations strongly suggest that vortioxetine has beneficial effects on depression-like behavior in rodents and that these latter may be mediated by direct activity at 5-HT receptors (19, 45). These effects could be related, for example, to desensitizing somatodendritic and activating postsynaptic 5-HT1A receptors (40).

#### Vortioxetine Improves Cognition in Healthy Animals and in Animal Models of Depression

A growing body of evidence from preclinical studies suggests that vortioxetine may also enhance cognitive functions, besides its antidepressant and anxiolytic effects (**Table 1**). For instance, it has been shown that this compound improves acquisition and retention of contextual fear memory, object recognition memory, and visuospatial memory in rats and mice. Mørk and collaborators (13) reported in healthy male Sprague–Dawley rats an improvement in the novel object recognition (NOR) test and an increase in freezing time during the retention test in a contextual fear-conditioning paradigm. In two other studies, in female Long–Evans rats, acute administration of vortioxetine restored memory performance deficits induced by 5-HT depletion (14, 15). This is in line with data obtained by Li and collaborators (18). They found that healthy middle-aged female mice exhibited impaired visuospatial memory in the novel object placement (location) (OP) test. Chronic treatment with vortioxetine, but not fluoxetine, improved visuospatial memory in these animals and increased hippocampal expression of several neuroplasticity-related genes (transcription and translation factors, genes involved in synaptic plasticity, signal transduction, and neurotransmission). Hippocampal growth factor levels, in contrast, were affected neither by vortioxetine nor by fluoxetine in this experiment (18). Memory improvements were also reported in the OP test (visuospatial memory) in healthy middle-aged mice after chronic treatment with vortioxetine (22). Surprisingly, the effects on memory were significant following a 1-month treatment (OP) but absent after a 3-month treatment (NOR, recognition memory) (22). The reasons for the lack of effect after the 3-month treatment might be due to the fact that a different behavioral task was used to evaluate the effects of vortioxetine on memory (1-month treatment: visuospatial memory vs. 3-month treatment: recognition memory).

**Table 1:** Studies exploring the cognitive effects of vortioxetine in mice and rats.

Authors	Animals	Treatment	Cognitive assessment	Main results
Mork et al., 2013 (13)	Healthy male Sprague–Dawley rats	1–10 mg/kg of vortioxetine administered subcutaneously 1 hour before the NOR or 1 hour before or immediately after acquisition of contextual fear conditioning.	Recognition (NOR)Contextual fear conditioning	Improvement in the NOR test. Increase in freezing time during the retention test in the contextual fear conditioning assessment.
du Jardin et al., 2014 (14)	Female Long–Evans rats with 5-HT depletion	Acute injections of vehicle or vortioxetine at 0.0001, 0.1, 3, or 10 mg/kg 1 hour prior to behavioral test.Chronic p.o. vortioxetine administration: *ad libitum* access to vortioxetine-infused food (0.6 g/kg of food weight) for 23 days.	Recognition (NOR)Spatial memory (SA)	Acute injection of vortioxetine dose-dependently reversed 5-HT depletion-induced memory deficits in both tests.Chronic p.o. vortioxetine administration significantly improved memory performance only in the NOR.
Jensen et al., 2014 (15)	Female Long–Evans rats with 5-HT depletion	Acute injections of vehicle or vortioxetine at 10 mg/kg 1 hour prior to behavioral test.	Recognition (NOR)Spatial memory (SA)	At similar SERT occupancies (> 90%) vortioxetine, but not escitalopram or duloxetine, restored memory performance deficits induced by 5-HT depletion.
Wallace et al., 2014 (16)	Male Sprague–Dawley rats with 5-HT depletion exposed to chronic intermittent cold stress	Acute injections of vehicle or vortioxetine at 10 mg/kg immediately prior to behavioral test (i.p.).Chronic p.o. vortioxetine administration: *ad libitum* access to vortioxetine-infused food (0.6 or 1.8 g/kg of food weight) for 10 days then restricted access (30 or 90 mg/kg per day) for 14 days.	Reversal learning (AS)	A single acute injection of vortioxetine significantly attenuated the 5-HT depletion-induced reversal learning impairment. Chronic intermittent cold stress impaired reversal learning. Chronic vortioxetine administration prevented the reversal-learning deficit.
Bétry et al., 2015 (17)	Male Sprague–Dawley OFA rats	Acute injections of vehicle or vortioxetine at 10 mg/kg immediately prior to behavioral test (i.p.).	Recognition (NOR)	Enhanced short-term episodic memory.Vortioxetine also prevented the effect of stress on hippocampal LTP and increased rapidly hippocampal cell proliferation.
Li et al., 2015 (18)	Healthy middle-aged C57BL/6 female mice (11 months old)	*Ad libitum* access to vortioxetine-infused food (0.6 g/kg of food weight) for 1 month.	Spatial memory (OP)	Chronic treatment with vortioxetine, but not fluoxetine, improved visuospatial memory and reduced depression-like behavior in the forced swim test.This was associated with change in the expression of multiple genes involved in neuronal plasticity. Vortioxetine did not reverse the age-associated decrease in stem cell proliferation in the hippocampus.
du Jardin et al., 2016 (19)	Male Flinders Sensitive Line (FSL) rats, a genetic model of depression, depleted of 5-HT	One injections of vehicle or vortioxetine at 10 mg/kg (i.p.) 1 hour (acute) or 24 hours (sustained) prior to behavioral test.	Recognition (NOR)	Vortioxetine, but not fluoxetine or S-ketamine, acutely ameliorated the memory deficits of FSL rats in the NOR irrespective of 5-HT tone (1 hour). No sustained effects were observed in the NOR (24 hours).
Pehrson et al., 2016 (20)	Male Wistar and Sprague–Dawley rats (adults and juveniles)	One injections of vehicle or vortioxetine at 0.1–10 mg/kg (s.c.) 1 hour (acute) prior to behavioral tests. Subchronic studies: food infused with 1.8 g vortioxetine per kg of food.	Social and object recognition memory Visual signal detection task	Acute vortioxetine reversed scopolamine-induced impairments in social and object recognition memory, but did not alter scopolamine-induced impairments in attention. Subchronic vortioxetine treatment failed to reverse scopolamine-induced social recognition memory deficits.
Hatherall et al., 2017 (21)	Male Sprague–Dawley rats exposed to chronic stress (intermittent cold stress, social defeat, immobilization, swim stress)	Vortioxetine was administered in the diet prepared to contain 0.33 g/kg chow corresponding to a dose of approximately 24 mg/kg per day.	Fear conditioning (with a tone; fear memory and extinction)	Chronic stress exaggerated the expression of conditioned fear memory. Vortioxetine restored fear memory to control levels and rendered extinction in stressed rats comparable with that in controls.
Li et al., 2017 (22)	Healthy middle-aged C57BL/6 female mice (12 months old)	*Ad libitum* access to vortioxetine-infused food (0.6 g/kg of food weight) for 1 or 3 months.	Spatial memory (OP)Recognition (NOR)	After 1-month treatment, vortioxetine improved visuospatial memory (OP) and reduced depression-like behavior. After 3 months, vortioxetine reduced depression-like behavior without affecting recognition memory (NOR).
Pehrson et al., 2018 (23)	Adult male Long–Evans and Sprague–Dawley rats; adult male C57BL/6NCrlBr mice	Acute vortioxetine doses ranged from 1 to 10 mg/kg (s.c.).Subchronic studies: food infused with 0.18–1.8 g vortioxetine per kg food	Attentional set-shifting test Spatial memory (OP)Recognition (NOR)	Acute or subchronic vortioxetine treatment attenuated phencyclidine-induced deficits in attentional set-shifting performance. Subchronic vortioxetine reversed phencyclidine-induced object recognition and object placement impairments in mice.
Felice et al., 2018 (24)	C57BL/6J Rj male mice; GFAP-thymidine kinase (TKC) male mice (for neurogenesis inhibition)	Vortioxetine was administered in the diet (1.8 g/kg of food weight; corresponding to ∼10 mg/kg) during 3–4 weeks.	Context discrimination	Vortioxetine improves context discrimination in mice through a neurogenesis independent mechanism.

AS, attentional set-shifting test; NOR, novel object recognition test; OP, novel object placement test; SA, Y-maze spontaneous alternation test.

Reversal learning, a form of cognitive flexibility, is impaired by chronic stress, a risk factor for depression, and the stress-induced impairment in reversal learning is sensitive to chronic selective serotonin reuptake inhibitor (SSRI) treatments and is mimicked by 5-HT depletion. It has been shown that chronic dietary administration of vortioxetine can restore reversal learning in animals whose capacity was damaged due to exposure to chronic intermittent cold (16). In accordance with these data, vortioxetine also prevents the effect of stress on hippocampal long-term potentiation (LTP), rapidly increases hippocampal cell proliferation (faster than fluoxetine), and enhances recognition memory in male Sprague–Dawley OFA rats (17). du Jardin and collaborators (19) also investigated the acute and sustained effects of S-ketamine (15 mg/kg), fluoxetine (10 mg/kg), or vortioxetine (10 mg/kg) on recognition memory in the NOR task. The investigators used Flinders Sensitive Line rats depleted or not of 5-HT. They found that vortioxetine, but not fluoxetine or S-ketamine, acutely ameliorated the memory deficits of Flinders Sensitive Line rats in the object recognition task irrespective of 5-HT tone (19). Hatherall and collaboratorss (21) have recently shown that chronic vortioxetine administration decreases the excessive expression of fear memory and the shift from active to maladaptive passive coping behavior induced by the chronic stress treatments: chronic plus acute prolonged stress and chronic unpredictable stress.

Furthermore, MDD figures as a risk factor for Alzheimer disease, since depressive symptoms significantly intensify the conversion of mild cognitive impairment into this dementia. Recently, Torrisi et al. (49) found that continuous treatment with either fluoxetine or vortioxetine might avoid cognitive deficits as well as depressive-like phenotype in a nontransgenic animal model of Alzheimer disease (induced by intracerebroventricular injection of amyloid-β (1–50)) with a key contribution of transforming growth factor β1.

Finally, Pehrson and collaborators (20) observed that acute administration of vortioxetine reverses scopolamine (an anticholinergic agent)–induced impairments regarding social and object recognition memory, without affecting scopolamine-induced impairments in attention. In this study, a modest and short-lived rise of hippocampal acetylcholine (ACh) levels was equally reported. Subchronic vortioxetine administration, on the other hand, neither reversed scopolamine-induced social recognition memory deficits, nor affected basal hippocampal ACh levels. In another study, the same team (23) demonstrated that either acute or subchronic vortioxetine administration attenuates phencyclidine (an *N*-methyl--aspartate [NMDA] receptor antagonist)–induced deficits in attentional set shifting performance and that both ondansetron (a selective 5-HT3 receptor antagonist) and escitalopram (a 5-HT reuptake inhibitor) can mimic this effect. Furthermore, subchronic vortioxetine reverses phencyclidine-induced object recognition and OP impairments in mice. More recently, Felice and collaborators (24) reported that vortioxetine improves context discrimination in mice. However, contrary to a previous hypothesis [cf. (17)], this improvement was observed through a neurogenesis-independent mechanism.

To sum up, in animal studies, vortioxetine appears to have beneficial effects on depression-related behavior and cognitive deficits that are distinct from those of SSRIs and serotonin-norepinephrine reuptake inhibitors (15, 19, 41, 44, 45). Altogether, these preclinical data provide further evidence of the therapeutic potential of vortioxetine in the management of depression disorders including depression-induced cognitive impairments in humans. The results from these experiments also suggest that multiple mechanisms could be at the origin of the procognitive properties of vortioxetine. Indeed, it has been shown that vortioxetine exerts, in addition to direct effects on serotonin, indirect effects on multiple neurotransmitters such as noradrenergic, histaminergic, cholinergic, GABAergic, and glutamatergic systems (demonstrated in both *in vitro* and *in vivo* studies) (10, 51, 52). Furthermore, a growing number of studies also indicate that vortioxetine modulates molecular targets that are related to neuroplasticity (53). For example, Waller and collaborators (54) have shown that neuroplasticity pathways, and protein-interaction networks are modulated by vortioxetine in rodents. They also found that chronic vortioxetine treatment in rodents modulates gene expression of neurodevelopmental and plasticity markers (55). Another example is that vortioxetine promotes maturation of dendritic spines *in vitro* in hippocampal cultures (56). In this study, vortioxetine, ketamine, and duloxetine induced spine enlargement. Nonetheless, increased number of spines in contact with presynaptic terminals was only observed following treatment with vortioxetine. Other mechanisms have been suggested, such as the facilitating effects of vortioxetine on LTP and on neurogenesis levels in the hippocampus (17, 24, 50). Vortioxetine, but not fluoxetine, also increases hippocampal brain–derived neurotrophic factor levels in healthy rats (57), as well as in rats subjected to chronic unpredictable mild stress (an animal model of depression) (58).

It is important to note that the studies described above have several limitations. For example, the behavioral effects reported are sometimes rather modest or appear only under particular conditions [e.g., social recognition is improved under acute dosing condition only (20)]. There is also a need for more systematic and comprehensive complementary studies testing the same behavioral parameters at short and long terms, under the same experimental conditions. Finally, a very high proportion of the studies cited in this section have been published by a small number of research teams, which are often from and/or sponsored by Lundbeck. Therefore, it would be useful to know if these data can be replicated and extended by independent studies.

#### Behavioral Effects of Vortioxetine: Involvement of the Tryptophan Metabolism?

There is a link between depression and the metabolism of tryptophan through kynurenine and serotonin pathways. Eskelund and collaborators (59) examined, in Sprague–Dawley and Flinders Sensitive Line rats, the effects of vortioxetine and other antidepressants (fluoxetine and ketamine) on various tryptophan metabolites in different brain regions and plasma. The effects of vortioxetine on tryptophan metabolites were also assessed in the cortical regions of lupus mice (MRL/MpJ-FasIpr), a murine model of increased depression-like behavior associated with inflammation. The investigators found that sustained vortioxetine and fluoxetine administration (at doses aimed to fully occupy serotonin transporters *via* food or drinking water for at least 14 days) decreased levels of the excitotoxin quinolinic acid independent of species. Ketamine, on the other hand, induced no significant effect.

Franklin and collaborators (60) studied the effects of vortioxetine and the SSRI paroxetine on pineal melatonin and monoamine synthesis in a subchronic tryptophan depletion model of depression based on a low tryptophan diet. Vortioxetine was administered *via* the diet (0.76 mg/kg of food weight) for 14 days. As results, tryptophan depletion-induced reductions of pineal melatonin and serotonin were reversed, while pineal noradrenaline was significantly increased (60). The authors suggested that the changes observed may be due to a strong 5-HT reuptake blocking action, along with possible additional effects on glutamate neurotransmission in the pineal gland *via* NMDA receptor modulation and with added impetus from increased noradrenaline output (60). In a subsequent study, the same team evaluated the effects of vortioxetine on potential biomarkers associated with tryptophan depletion including serum aldosterone, corticosterone, and interleukin 6 levels together with indirect indicators of glutamate neurotransmission. As in the previous study, vortioxetine was administered *via* the diet (10 mg/kg per day) for 14 days. Vortioxetine reversed tryptophan depletion-induced depressive-like behavior and reduced tryptophan depletion-induced increases of serum corticosterone, aldosterone, IL-6, and NMDA and α7-nicotinic ACh receptor expression in the amygdala and hippocampus, respectively (43). Since paroxetine, an SSRI, had almost no impact in this animal model of depression, the observations reported in the two studies mentioned above confirm the hypothesis that vortioxetine’s antidepressant activity may involve mechanisms beyond SERT inhibition (43, 60). While the effect of vortioxetine on tryptophan metabolism may be at the origin of its antidepressant effect, it is not clear yet whether this potential mechanism of action may be linked to its procognitive effects.

### Human Studies

#### Efficacy of Vortioxetine on Cognitive Functioning: Clinical Data

The short-term effects of vortioxetine 5 to 20 mg/day on cognitive performance in adults with current MDD have been investigated in several cognitive domains, notably psychomotor speed and executive function [Digit Symbol Substitution Test (DSST), Trail Making Tests (TMT) A and B, Simple Reaction Time], acquisition and memory [Rey Verbal Learning Test (RAVLT)], attention and cognitive control (Stroop task), and with secondary subjective measures of cognitive function [Perceived Deficits Questionnaire (PDQ)] (**Table 2**).

**Table 2:** Randomized controls and open-label trials exploring the cognitive effects of vortioxetine in human.

Authors	Design	Population	Treatment (number of patients)	Cognitive assessment	Main results
Katona et al., 2012 (25)	Double-blind RCT 8 weeks	MDD > 3 months Aged ≥65 yr MADRS ≥26	Vortioxetine 5 mg/day (156) *vs.* Duloxetine 60 mg/day (151) *vs.* Placebo (145)	DSST RAVLT RAVLT	Significant improvement in DSST and RAVLT Vortioxetine > placebo for DSST and RAVLT Duloxetine > placebo only for RAVLT
McIntyre et al., 2014 (26)	Double blind RCT 8 weeks	MDD > 3 months Aged ≥18 and ≤65 yr MADRS ≥26	Vortioxetine 10 mg/d (192) *vs.* Vortioxetine 20 mg/day (204) *vs.* Placebo (194)	DSST RAVLT TMT-A TMT-B SRT CRT Stroop test PDQ	Vortioxetine > placebo for all measures with the exception of CRT for vortioxetine 20 mg Effect on cognitive function independent of mood improvement
Mahableshwarkar et al., 2015 (27)	Double blind RCT 8 weeks	MDD > 3 months MADRS ≥26 Aged ≥18 and ≤65 yr	Vortioxetine 10 or 20 mg/day (168) *vs.* Placebo (164) *vs.* Duloxetine 60 mg/day (176)	DSST TMT-A TMT-B Stroop Test Groton Maze Learning Test Detection Task Identification Task One-Back Task PDQ CPFQ	Vortioxetine > placebo for DSST, TMT-A, PDQ, attention/concentration and planning/organization subscores of PDQ Vortioxetine = duloxetine for DSST Vortioxetine = placebo for CPFQ
Harrison et al., 2016 (28)	Double blind RCT 8 weeks	MDD > 3 months Aged ≥18 and ≤65 yr MADRS ≥26	Vortioxetine 10 mg/day (192) *vs.*Vortioxetine 20 mg/day (204) *vs.* Placebo (194)	DSST RAVLT TMT-A TMT-B Stroop test SRT CRT	At 8 weeks, vortioxetine 10 mg > placebo for executive function, attention/speed of processing, memory, and DSST after correcting for change in depression severity At 8 weeks, vortioxetine 20 mg > placebo for executive function and DSST At 1 week, vortioxetine 10 mg > placebo for executive function, attention/speed of processing, DSST number of correct symbols
McIntyre et al., 2017 (29)	Post hoc analysis of 5 RCT at 8 weeks	2,206 MDD MADRS ≥26 Working (1, 254) vs. nonworking patients	Vortioxetine 10 mg/day *vs.* Vortioxetine 20 mg/day *vs.* Placebo	DSST TMT-A TMT-B Stroop test SRT CRT PDQ	Vortioxetine > paroxetine for DSST, Stroop, TMT, SRT, PDQ Superior effect on measures of cognitive functioning more pronounced in patients with a professional type of position.
Baune et al., 2018 (30)	Double blind RCT 8 weeks	MDD > 3 months Employed patients Aged ≥18 and ≤65 yr	Vortioxetine 10 mg/day (48) *vs.* Paroxetine 20 mg/day (54) *vs.* Placebo (48)	DSST TMT-A TMT-B Stroop test SRT CRT PDQ	Vortioxetine = paroxetine = placebo for DSST Vortioxetine = paroxetine = placebo for UPSA-B Vortioxetine > paroxetine for composite cognition *z* score Change in composite cognition *z* score positively correlated with changes in UPSA-B total score Change in composite cognition *z* score negatively correlated with changes in FAST and MADRS scores Change in PDQ-D positively correlated with changes in FAST and MADRS total scores
Vieta et al., 2018 (31)	Double blind RCT 8 weeks	MDD Aged ≥18 and ≤65 yr MADRS ≥22 Inadequate response to at least 6 weeks of SSRI or SNRI monotherapy	Vortioxetine 10–20 mg/day (50) *vs.* Escitalopram 10–20 mg/day (49)	DSST TMT-A TMT-B Stroop test RAVLT SRT CRT PDQ	Vortioxetine = escitalopram
Freeman et al., 2017 (32)	Open-label study 8 weeks	21 early postmenopausal and perimenopausal women with MDD	Vortioxetine	DSST CPFQ Cogstate test	Improvement in DSST and CPFQ No improvement in Cogstate test scores
Smith et al., 2018 (33)	Double blind RCT 2 weeks	Remitted depressed (48) Healthy volunteers (48)	Vortioxetine 20 mg/day *vs.* Placebo	N-back working memory task RAVLT TMT PDQ	Vortioxetine: improvement in TMT-A and PDQ Vortioxetine reduced activation in the right dorsolateral prefrontal cortex and left hippocampus
Levada and Troyan, 2019 (34)	Open-label study 8 weeks	MDD Aged ≥18 and ≤65 yr MADRS ≥7	Vortioxetine (36) *vs.* Escitalopram (20)	DSST RAVLT TMT-B	Vortioxetine > escitalopram for RAVLT
Nierenberg et al., 2019 (35)	Double blind RCT 8 weeks	MDD (151) Aged ≥18 and ≤65 yr HDRS ≤10PDQ >25	Current SSRI + placebo *vs.* SSRI + vortioxetine 10–20 mg/day *vs.* Vortioxetine 10–20 mg/day + placebo.	DSST RAVLT acquisition and delayed recall, TMT-A and B, SRT CRT STROOP congruent and incongruent	Improvement of cognitive performances in the three groups No statistical differences
Yan et al., 2019 (36)	Double blind RCT	MDD (81)	Vortioxetine 10 mg (41) *vs.* Vortioxetine 10 mg + CBT (41)	WCST CPT	Vortioxetine+ CBT > vortioxetine for CPT scores Vortioxetine+ CBT > vortioxetine for DSST Higher levels of BDNF in the serum in the vortioxetine + CBT group
Chokka et al., 2019 (37)	Open-label study 52 weeks	MDD (199) QIDS ≥15 PDQ ≥30	Vortioxetine (10–20 mg/day)	PDQ	Improvement in cognitive symptoms Association between improvements in cognitive symptoms and workplace productivity loss after 12 weeks and 52 weeks of treatment

CBT, cognitive behavioral therapy; CPFQ, Cognitive and Physical Functioning Questionnaire; CRT, choice reaction time; dlPFC, dorsolateral prefrontal cortex; DSST, Digit Symbol Sign Test; FAST, Functioning Assessment Short Test; MDD, Major depressive disorder; MADRS, Montgomery-Åsberg Depression Rating Scale; PDQ, Perceived Deficit Questionnaire; QIDS, Quick Inventory of Depressive Symptomatology–Self-report; RAVLT, Rey Auditory Verbal Learning Test; SRT, simple reaction time; TMT-A or -B, Trails Making Test; UPSA-B, San Diego Performance-Based Skills Assessment; WCST, Wisconsin Card Sorting Test.

In a double-blind placebo-controlled study, Katona et al. (25) randomized 453 elderly MDD patients to receive vortioxetine 5 mg/day, duloxetine 60 mg/day, or placebo. Vortioxetine separated from placebo in both the DSST and the RAVLT (standardized effect sizes 0.25 for DSST, 0.27 for RAVLT acquisition, and 0.24 for RAVLT delayed recall), indicating improvements in processing speed, verbal learning, and recall domains. Duloxetine failed to improve DSST scores, suggesting that vortioxetine exerts its beneficial effect on more cognitive domains. Moreover, a *post hoc* path analysis revealed that more than two-thirds of vortioxetine’s impact on cognition is attributable to direct treatment effect, not to improvement in severity of depressive symptoms, indirectly, as with duloxetine.

Concordant results were obtained in another double-blind randomized controlled trial (RCT) with a fixed dose of vortioxetine (10–20 mg/day) in younger MDD patients (26). In comparison to placebo, vortioxetine at both doses was significantly superior in terms of improvement of a weighted composite score of DSST, RAVLT acquisition, and RAVLT delay at 8 weeks, with mean treatment differences in the composite cognition score versus placebo of 0.36 (vortioxetine 10 mg/day, *P* < 0.0001) and 0.33 (vortioxetine 20 mg/day, *P* < 0.0001). Path analyses showed that this effect was largely a direct treatment effect at both doses (vortioxetine 10 mg: 64% and vortioxetine 20 mg: 48%) independent of improvements in overall depression severity. In secondary analyses evaluating objective cognitive measures as well as patient-reported outcomes, vortioxetine at both doses also showed superiority versus placebo, suggesting a favorable effect on memory, concentration, and planning/organization. It is important mentioning that the higher dose of vortioxetine resulted in a greater improvement of Montgomery–Åsberg Depression Rating Scale (MADRS) total scores. This dose–response effect was not observed for the cognitive performance scores, suggesting that vortioxetine exerts its procognitive and antidepressant actions *via* separate mechanisms. In a duloxetine-referenced trial, Mahableshwarkar et al. (27) explored the effects of flexible doses of vortioxetine (10–20 mg/day) on objective and subjective measures of cognitive functions and on functional capacity in patients with self-reported cognitive dysfunction. While both antidepressants significantly improved attention/concentration and planning/organization PDQ scores, only vortioxetine significantly improved DSST and TMT-B performance and the University of San Diego Performance-Based Skills Assessment (UPSA) scores, indicating restored executive function and functional capacity as well as faster cognitive processing. Path analysis has indicated that the effect of vortioxetine on cognitive performance is largely independent of the alleviation of depressive symptoms, while more than one-half of the duloxetine effect on cognitive deficits is attributable to improvements in depressive symptoms. Harrison et al. (61) studied which specific domains vortioxetine improves and demonstrated a procognitive effect in vortioxetine-treated patients at 8 weeks, even after correcting for a change in depression severity. In this study, vortioxetine at 10 and 20 mg/day separated from placebo for executive function, attention/speed of processing, and memory from the first week of treatment, indicating that vortioxetine may exert its positive effect on cognition early. It was stressed that this outcome does not inevitably reflect an enhancement of one specific domain, but mostly an improvement in a number of cognitive skills engaged by the DSST, as a result of changes in the other cognition variables. Interestingly, in a *post hoc* analysis of data from five RCTs, McIntyre et al. (29) reported that the positive effects of vortioxetine on both objective (DSST, TMT) and subjective (PDQ) measures of cognition were more prominent in patients with MDD who were active at work (especially in manager/administrator positions) than in the total population of those trials. This highlights the potential of vortioxetine to diminish cognitive dysfunction and enhance functional capacity, principally in working patients with MDD.

In primary analyses of their RCT including paroxetine as an active reference, Baune et al. (39) evaluated the effects of vortioxetine on cognitive function in a sample of employed depressed patients. They observed no statistically significant effects on cognitive performance or functional capacity, as measured by DSST and UPSA-B, respectively, after 8 weeks of pharmacological treatment versus with placebo. However, they noted a similar reduction in depressive symptoms associated with an improvement in cognition following pharmacological treatments. Moreover, a tendency of numerical differences in favor of vortioxetine was perceived across seven secondary cognitive endpoints. More recently, Vieta et al. (31) observed improvements in DSST and UPSA-B performance in a small sample of patients with inadequate response to SSRI or SNRI (selective noradrenalin reuptake inhibitor) monotherapy treated with flexible doses of either vortioxetine or escitalopram. Although no statistically significant differences were reported, vortioxetine was superior to escitalopram in terms of improvement of the number of DSST correct symbols, with a mean difference between the treatments of 2.0 points in favor of vortioxetine. Further, a trend of numerical differences in favor of vortioxetine was seen across seven secondary cognitive endpoints.

In a randomized, double-blind study, Nierenberg et al. (35) assessed the efficacy of vortioxetine as an adjunctive treatment to SSRI and as a monotherapy versus continued SSRI in the treatment of residual cognitive symptoms in the remitted phase of MDD. One hundred fifty-one patients with MDD who achieved full or partial remission with SSRI, but reported residual cognitive symptoms, were randomized to receive 8 weeks of current SSRI + placebo, SSRI + vortioxetine (10–20 mg/day), or vortioxetine (10–20 mg/day) + placebo. From baseline to week 8, all treatment groups improved performance in objective measures of executive function, attention, processing speed, learning, and memory, with statistically nonsignificant treatment differences. These improvements had the tendency of being numerically larger when vortioxetine monotherapy was compared with SSRI monotherapy, as well as to display a better numerical performance regarding further improvement of depressive symptoms with vortioxetine as adjunctive treatment. Another study recently explored the effect of vortioxetine in a combination therapy. Yan et al. (36) reported that vortioxetine combined with cognitive behavior therapy leads to greater improvements in executive functions assessed with the Wisconsin Card Sorting Test and attention function assessed with the Continuous Performance Test compared to vortioxetine alone. Moreover, the level of brain-derived neurotrophic factor in the serum of patients treated with vortioxetine combined with CBT was higher than that in the group treated with vortioxetine alone.

Besides RCTs, three open-label trials examined the cognitive benefits of vortioxetine. Freeman et al. (32) reported an improvement in DSST scores and in the Cognitive and Physical Functioning Questionnaire after treatment with a flexible dose of vortioxetine in the context of early postmenopausal and perimenopausal depression in 24 women (32). In an escitalopram-referenced trial, Levada and Troyan (62) specifically assessed the effects of vortioxetine on cognitive impairments and global, working, social, and family functioning in a cohort of 56 MDD patients. At week 8, both antidepressants exhibited procognitive effects. However, the effects of vortioxetine on short-term (working) memory and executive disturbances, attention, and processing speed were significantly higher. The impact of vortioxetine was also significantly superior on working, social, family, and total functioning. Finally, an open-label, real-world study was undertaken to examine the association between cognitive symptoms and workplace productivity in working patients with MDD treated with vortioxetine (37). Along the 52 weeks of treatment, significant improvements in cognitive symptoms, work productivity, and functional outcomes were reported. Interestingly, the enhancements in patient-rated cognitive symptoms could predict long-term improvements in functional outcomes, even following adjustment for reduction in depressive symptoms.

Several meta-analyses pooling available data regarding the cognitive effects of vortioxetine have yielded concordant results (**Table 3**). Focusing on the DSST in a meta-analysis of five RCTs, McIntyre et al. (28) found that fixed or flexible dosages of vortioxetine (10–20 mg/day) were more effective than placebo and duloxetine, with beneficial effects independent of the improvement of depressive symptoms as assessed by MADRS. The effect sizes derived from this meta-analysis were small in magnitude after adjustment for MADRS (standard mean difference with placebo was 0.24 and 0.19 with duloxetine, respectively). Rosenblat et al. (38) included 17 controlled trials in their meta-analysis, aiming to assess the pooled efficacy of antidepressants on several cognitive domains in MDD. Seven antidepressants were assessed in the context of a placebo-controlled trial, and apparently, vortioxetine had the largest pooled effect size on psychomotor speed, executive control, and cognitive control. Duloxetine, alternatively, displayed the greatest effect on delayed recall.

**Table 3:** Meta-analysis exploring the cognitive effects of vortioxetine in human.

Authors	Design	Population	Treatment(number of patients)	Cognitive assessment	Main results
McIntyre et al., 2016 (28)	Meta-analysis of 3 RCTs	Current MDD Aged ≥18 yr. MADRS ≥ 26	Vortioxetine 5–20 mg/d *vs.* Duloxetine 60 mg/d *vs.* Placebo	DSST	Vortioxetine > placebo for DSST after correcting for change in depression severity Vortioxetine > duloxetine for DSST after correcting for change in depression severity
Rosenblat et al., 2015 (38)	Meta-analysis of 17 RCTs	2,550	Vortioxetine (728) Duloxetine (714) Paroxetine (23) Citalopram (84) Phenelzine (28) Nortryptiline (32) Sertraline (49) Placebo	DSST or combined speed TMT-B; RAVLT; Stroop	Vortioxetine > other antidepressants for DSST
Baune et al., 2018 (39)	Network meta-analysis of 12 RCTs	MDD (3, 738)	Vortioxetine (725) Duloxetine (707) Citalopram (84) Escitalopram (54) Fluoxetine (127) Sertraline (240) Phenelzine (28) Desipramine (9) Nortriptyline (102)	DSST	Vortioxetine > placebo for DSST Vortioxetine > other antidepressants for DSST

CRT, choice reaction time; DSST, Digit Symbol Sign Test; MDD, major depressive disorder; MADRS, Montgomery-Åsberg Depression Rating Scale; PDQ, Perceived Deficit Questionnaire; RAVLT, Rey Auditory Verbal Learning Test; SRT, simple reaction time; TMT-A or -B, Trails Making Test; UPSA-B, San Diego Performance-Based Skills Assessment.

Similarly, Baune et al. (39) reported that vortioxetine improves both objective and subjective measures of cognitive function, independently of its effect on depressive symptom severity. This network meta-analysis of 12 RCTs using the DSST showed that vortioxetine was the only antidepressant that improved cognitive dysfunction in the DSST *versus* placebo [standardized mean difference: 0.325 (95% confidence interval = 0.120–0.529, *P* = 0.009)]. Moreover, subjectively experienced cognitive function significantly improved in the vortioxetine treatment group, as measured by the PDQ; equally, each of the subdomain scores (i.e., planning/organization, attention/concentration, prospective and retrospective memory) displayed a significant enhancement.

#### Efficacy of Vortioxetine on Cognitive Functioning: Neuroimaging Data

Only one study aimed to evaluate the ability of vortioxetine to target directly the neural circuitry implicated in the cognitive deficits in depression and its capacity to mitigate those deficits (33). In this study with a double-blind design, Smith et al. (33) sought to determine the effect of treatment on functional magnetic resonance imaging responses during an N-back working memory task and on neuropsychological measures of executive function, speed and information processing, attention, and learning and memory. They reported that when compared to placebo, 14 days of treatment with 20 mg vortioxetine significantly reduced activity in the right dorsolateral prefrontal cortex and left hippocampus as well as across a network of temporal–parietal areas during the N-back task. Interestingly, these effects were dissociated from the mood effects of the drug. These neural changes were accompanied by improved subjective ratings of cognitive function as measured by the PDQ and improved performance in the TMT-A task. These authors suggest that vortioxetine may facilitate the ability to switch off the default-mode network and encourage more efficient recruitment of task-positive networks during working memory tasks. This could explain the direct and independent effect of vortioxetine on cognition, specifically on executive function and working memory.

## Discussion

This review aimed to offer an overview of the effect of vortioxetine on cognitive function in depression.

Preclinical studies offer a rationale to understand the molecular mechanisms of vortioxetine’s cognitive effects and provide a basis for the differentiation of vortioxetine from conventional antidepressants with respect to effects on cognitive functioning. Overall, in animal models, acute, subchronic, and chronic administration of vortioxetine exerts positive effects across a range of cognitive tasks (13–24). Vortioxetine achieved enhancements in acquisition and retention of contextual fear memory, object recognition memory, and visuospatial memory in rats and mice. Furthermore, pharmacological studies in some of the animal models indicate that the cognitive benefits of vortioxetine rely on its action on receptor activity, in particular 5-HT3 receptor antagonism (63).

GABA, the main inhibitory neurotransmitter in the brain, exerts inhibitory action on the glutamatergic excitatory neurotransmission system and key regulators of cognitive processing (64). The blockade of 5-HT3 receptor–expressing GABAergic interneurons by vortioxetine increases glutamate-dependent synaptic neurotransmission and enhances firing of pyramidal neurons (65, 66). Enhanced release of glutamate in the prefrontal cortex and hippocampus enhances LTP (a cellular mechanism of learning and memory (67)) and promotes maturation of dendritic spines as shown *in vitro* in hippocampal cultures (56). Interestingly, *in vivo* studies of the rat prefrontal cortex showed that effects on modulation of GABAergic and glutamatergic neurotransmission occur at doses across the clinically relevant dose range (5–20 mg/day) (50, 66, 68).

The stimulation of 5-HT1A receptors further disinhibits pyramidal neurons, while partial agonism of 5-HT1B receptors by vortioxetine leads to greater glutamate and 5-HT release. Downstream, 5-HT modulates the release of several neurotransmitters including dopamine, noradrenaline, ACh, and histamine (69). This is significant from a cognitive perspective as ACh is involved in learning and memory, and histamine is implicated in attention, alertness, and memory (70, 71). The involvement of tryptophan metabolism and the modulation of LTP as well as the increase in neurogenesis levels and brain-derived neurotrophic factor levels in the hippocampus could also potentially explain vortioxetine’s unique procognitive action (60, 68). The distinctive serotoninergic properties of vortioxetine in the hippocampus and prefrontal cortex, in comparison to SSRIs that stimulate every 5-HT receptor, are likely an important feature of its cognitive activity (69). Of note, microdialysis studies in rats have shown that vortioxetine induces an approximately twofold higher rise in extracellular 5-HT concentration in comparison to an SSRI. In humans, PET studies using 5-HT transporter ligands to quantify the 5-HT transporter occupancy in the brain across different dose levels indicate that the mean 5-HT transporter occupancy is approximately 50% at 5 mg/day and 65% at 10 mg/day and increases to above 80% at 20 mg/day (34, 72). While the occupancy of 5‐HT transporter should be around 80% for a conventional SSRI/SNRI to be therapeutically useful, these studies suggest that vortioxetine may be efficacious at lower 5‐HT occupancy than necessary with SSRI/SNRIs (73). Taken together, the results of these studies offer a scientific basis for the differentiation of vortioxetine from SSRI and SNRI antidepressants with respect to effects on cognitive functioning and a rationale for continued studies to understand the underlying molecular mechanisms of vortioxetine in greater detail.

In addition to its antidepressant effects, vortioxetine has demonstrated cognitive-enhancing properties in humans in seven short-term RCTs (6–12 weeks) (26–35), three meta-analyses (28, 30, 38), and one open-label trial (62), wherein cognitive function was the primary outcome of interest. At this timepoint, significant improvements have been reported in processing speed, verbal learning, and recall domains in young and elderly adults with recurrent, moderate to severe MDD. These improvements, independent of the alleviation of depressive symptoms, represent a direct effect of vortioxetine and have been reported at doses across the clinically relevant dose range (5–20 mg/day) without a dose–response effect (26), suggesting that vortioxetine exerts its procognitive and antidepressant actions *via* separate mechanisms. A more important procognitive effect was observed in the subpopulation that was working at study baseline, independently of the effects on depressive symptom relief. Across five placebo-controlled studies, vortioxetine recipients also showed a significant improvement in subjective measures of cognitive function and a significant and clinically meaningful effect in an objective measure of functional capacity (5, 27, 29–31). Given the significant relationship between functional impairment and cognitive dysfunction, these studies appear to add to the multiple evidence indicating that vortioxetine positively influences both symptomatic and functioning outcomes.

Meta-analyses comparing the effects of different classes of antidepressants, including SSRIs and SNRIs, indicate that conventional antidepressants may to a degree improve cognitive deficits associated with MDD, although the majority of supporting studies have limitations such as small sample sizes, lack of placebo controls, or an absence of prespecification of cognition as a primary endpoint (28, 30, 38). They also demonstrate that vortioxetine distinguishes itself from other antidepressants in terms of effects on cognitive function, probably in line with its specific action on brain substrates relevant to cognitive function. Vortioxetine had the greatest effects on DSST scores, but also on psychomotor speed, executive control, and cognitive control among all antidepressants evaluated for cognitive effects, except in Rosenblat and colleagues’ (38) meta-analysis in which duloxetine had the greatest effect on delayed recall. The different mechanisms of action of the two compounds could mediate these differential effects described. As discussed previously, the rapid and sustained increase of 5-HT neurotransmission produced by vortioxetine could not be attained with an SSRI, particularly in brain regions such as the prefrontal cortex and ventral hippocampus, known to be clinically relevant for cognition. The results of these studies thus indicate that vortioxetine could be a useful option to treat cognitive dysfunction in depressed patients, as a first-line treatment, or for patients who reached full or partial remission with an SSRI but still report residual cognitive symptoms.

Although the effects of vortioxetine on cognition seem promising, several limitations need to be addressed. Methodological difficulties and a lack of long-term studies are confounding results across the field. These, together with variability in study design and in cognitive outcome measures, are limitations that must be overcome. Especially in meta-analyses, high variability is observed between the trials included in reporting of cognitive outcomes, patient numbers, timepoints of assessments, and outcomes, which limit the generalizability of the results and caution the interpretation. In three studies, results indicate numerical benefits with vortioxetine in cognitive performance, with statistically nonsignificant differences with the active comparator (35–39). Their small sample sizes and consequently lower statistical power may explain the inability to detect treatment differences. Only five studies included in this review used active comparators in assessing vortioxetine efficacy, and cognition was not systematically the primary endpoint (25, 32, 36, 37). Furthermore, one of the source trials only required patient-reported measures of cognition at baseline. Although it has been proposed that this reporting system may be more applicable to clinical practice, it will be important for future trials to investigate objectively measured deficits at inclusion for comparison (27). The phase of MDD, namely, subsyndromal, acute clinical, or postacute, also requires consideration. Surprisingly, only one RCT investigated the effects of the interventions on cognition in a remitted state of MDD (35). In addition, no study revealed whether the positive effect of vortioxetine on cognition represents a return to premorbid levels for these participants. The general paucity of such trials severely limits inferences about the causality of the effects of the interventions on depressive and cognitive symptoms. It should also be noted that a small number of studies attempted to assess the impact of long-term treatment with vortioxetine on cognition. Several trials focused on the DSST, a measure of information processing speed, executive function, and attention as a primary endpoint and on the RAVLT, which assesses learning and memory. As pointed out by Harrison et al. (61), it is noteworthy that usually individual cognitive tests are labeled as measures of specific domains, while in reality they index multiple cognitive skills. Interestingly, the data point out a lack of evidence on engagement of specific targets with particular cognitive domains. Vortioxetine seems in fact to enhance performance in diverse cognitive domains. Lastly, a small number of studies were from and/or sponsored by Lundbeck, which represents a significant limitation (36, 47, 48).

Future research could explore the efficacy of vortioxetine to mitigate cognitive dysfunction in MDD in combination with treatment specifically designed to target cognitive performance. Indeed, a combination of pharmacological interventions and psychological approaches may be the most effective treatment both in terms of acute response and relapse prevention in MDD, but also in targeting cognitive dysfunction. For example, combining vortioxetine with cognitive behavioral therapy could be a direction to explore to enhance cognitive functioning in MDD. An additional opportunity worth exploring would be to combine vortioxetine with brain stimulation techniques such as transcranial magnetic stimulation or transcranial direct current stimulation or with other psychotropic agents with known beneficial effects in the domain of cognition (e.g., psychostimulants). Of interest, in line with recent findings suggesting that the targeting of inflammatory signaling pathways is an interesting strategy to improve the responsiveness of unipolar depressive patients, an RCT conducted by Fourrier and colleagues (74) will explore the effects of vortioxetine as an add-on strategy with 400 mg celecoxib, an anti-inflammatory COX-2 inhibitor, on cognitive function and inflammatory markers. Finally, more work is needed to better specify the cognitive effects of vortioxetine and possibly identify patient subgroups to whom treatment would be most beneficial. Additionally, long-term studies are also required toward a better understanding on the effects of cognitive dysfunction treatment on long-term outcomes of MDD, such as functional recovery and development of dementia or other cognitive disorders. Interestingly, a recent RCT investigated the efficacy of vortioxetine on cognitive functions in Alzheimer disease patients with depressive symptoms and reported significant improvement in most of the cognitive tests applied (75).

It would also be useful to integrate extensive examination of distinct cognitive functions such as the processing of information with emotional valence in human studies, as positive effects of vortioxetine have been reported in the affective bias test in rat (48). This is of particular importance as numerous studies emphasize the importance of negative biases in information processing in the etiology and maintenance of depressive disorders (76). Finally, it would be particularly interesting to evaluate vortioxetine’s effect on neural circuit/subcircuit connectivity to understand its mechanisms of action in greater detail.

Despite research advances recognizing cognition as an important target, treatment advances are still unsatisfactory due to methodological difficulties across the field. Vortioxetine constitutes a promising therapeutic alternative in the presence of cognitive dysfunction, and its impact on functional capacity and its good safety/tolerability profile argue in favor of its use. Furthermore, the superior effect observed in working patients suggests additional advantages in terms of psychosocial functioning, as compared with more sedating antidepressant drugs. The actual mechanism responsible for its action still remains to be fully elucidated.

## Author Contributions

DB and VVW conducted the literature search. DB, EH, and VVW contributed substantially to writing of the article or revising it critically for important intellectual content and to final approval of the version to be submitted.

## Conflict of Interest

The authors declare that the research was conducted in the absence of any commercial or financial relationships that could be construed as a potential conflict of interest.
